# Pelvic organ prolapse is highly prevalent in women with spina bifida

**DOI:** 10.1002/bco2.70113

**Published:** 2025-11-24

**Authors:** Alexandre Dubois, Briac Malandain, Juliette Hascoet, Camille Haudebert, Charlène Brochard, Claire Richard, Caroline Voiry, Krystel Nyangoh Timoh, Andrea Manunta, Emmanuelle Samson, Benoit Peyronnet

**Affiliations:** ^1^ Department of Urology University of Rennes Rennes France; ^2^ French referral network of Spina Bifida Rennes University Hospital Rennes France; ^3^ Department of Gastro‐Enterology University of Rennes Rennes France; ^4^ Department of Physical Medicine and Readaptation University of Rennes Rennes France; ^5^ Department of Obstetrics and Gynecology University of Rennes Rennes France

**Keywords:** childbearing, neurogenic, pelvic organ prolapse, spina bifida, urinary incontinence

## Abstract

**Introduction:**

Women with spina bifida often experience neurological impairments leading to pelvic organ dysfunction, including difficulties with bladder and bowel emptying that necessitate frequent Valsalva manoeuvres. These factors, combined with pelvic floor weakness, may increase the risk of pelvic organ prolapse (POP). This study aimed to assess the prevalence of POP in women with spina bifida, identify associated risk factors and evaluate outcomes of surgical management.

**Methods:**

We retrospectively analysed a prospectively maintained database of women with spina bifida seen at a French referral centre from 2007 to 2024. Age under 18 and congenital perineal abnormality were exclusion criteria. The primary outcome was the presence of POP grade 2 or higher (Baden‐Walker classification). Secondary outcomes included symptomatic POP requiring surgery, recurrence after surgery, use of vaginal pessaries and related symptoms.

**Results:**

POP grade ≥2 was present in 14.8% of patients. Women with POP were older (median 44 vs. 31 years; p < 0.0001) and more frequently parous (58.5% vs. 18.3%; p < 0.0001), although 41.5% of POP cases occurred in nulliparous women. Apical prolapse was predominant (64.3%). Among 11 patients who underwent POP surgery, 54.5% experienced recurrence. Multivariate analysis identified parity (OR 5.33; p = 0.005) and lower maximum urethral closure pressure (OR 0.97; p = 0.02) as independent risk factors.

**Conclusions:**

POP is highly prevalent in young adult women with spina bifida, including many nulliparous patients. The parity status and a low maximum urethral closure pressure could be associated with an increased risk of POP in this population. High recurrence after surgery highlights the need for information, routine screening and tailored management in this population.

## 1 INTRODUCTION

Although spina bifida is considered a rare condition, its prevalence is about 1 in 10 000 live births in advanced countries.^1^, ^2^ The neurological impairments associated with spina bifida are usually not complete and tend to combine features of central and peripheral nervous system alterations.^3^ This results in a high prevalence of pelvic organ dysfunctions, including flaccid perineal paralysis, detrusor acontractility and bowel dysfunction or dyschesia.^4^, ^5^, ^6^

These features may determine a higher occurrence of pelvic organ prolapse (POP) in spina bifida patients due to the frequent use of Valsalva manoeuvre multiple times per day to empty their bladder or pass stools. This repeated increase in intra‐abdominal pressure associated with reduced pelvic floor resistance, represents a theoretical risk factor for POP. However, while childbirth—one of the main determinants of POP—is historically less prevalent in adult women with spina bifida compared to the general population, recent years have shown an increasing birthrate in this population.^7^ Despite this trend, there is limited data in the literature regarding the risk of POP in patients with spina bifida, and no study to date has specifically aimed to identify their risk factors. The aim of the present study was to assess the prevalence of POP in women with spina bifida, to identify associated risk factors and to evaluate the outcomes of their surgical management.

## 2 METHODS

### 2.1 Study design

After institutional review board approval (CNIL 1412467), a retrospective analysis of the prospectively maintained database of the French referral centre for spina bifida was conducted.

The charts of all female patients with spina bifida seen at the centre between September 2007 and 2024 were reviewed. Exclusion criteria included: (i) patients younger than 18 years of age and (ii) the presence of associated congenital perineal anomalies.

All medical records were reviewed to document a detailed clinical assessment of pelvic organ prolapse and the following baseline characteristics were recorded for each patient in a dedicated computerized dataset: age at the time of evaluation; body mass index; type of occupation; bladder management (clean‐intermittent catheterization [CIC] vs. spontaneous voiding vs permanent drainage); type of spinal dysraphism (open vs. closed, other); the level of neurological impairment (categorized as sacral, lumbar or thoracic); the mobility status (wheelchair user vs. ambulator); the parity status (nulliparous, history of previous vaginal delivery and/or caesarean section); history of augmentation cystoplasty or urinary diversion; history of previous anti‐incontinence procedure—and use of antimuscarinic drugs.

### 2.2 Neurological assessment

The type of spinal dysraphism (open vs. closed) was determined based on prior spinal imaging and/or operative reports, as well as a magnetic resonance imaging (MRI) scan of the spine, which was routinely performed for all patients during their first visit to the centre. The American Spinal Injury Association Impairment Scale (AIS) was used to assess neurological function. Based on clinical evaluation, the sensorimotor level of neurological impairment was classified as sacral, lumbar or thoracic.

### 2.3 Gastroenterological assessments and follow‐up

At their initial visit to the French national referral centre for spina bifida, all patients underwent a comprehensive gastrointestinal assessment, as previously described.^8^ Bowel function was evaluated through a standardized clinical interview. A physical examination was performed in the lithotomy position to assess for pelvic organ prolapse, following previously established methods, with additional assessment for rectal prolapse. Rectal prolapse was classified as either rectal intussusception or external rectal prolapse. A digital rectal examination (DRE) was systematically included as part of the evaluation. Gastroenterological follow‐up included repeat assessments encompassing all previously described steps, performed every 1 to 3 years based on the severity of bowel dysfunction. In patients with a diagnosis of rectal intussusception, follow‐up included repeat defecography.

### 2.4 Initial urological assessment and follow‐up

During their initial visit to the referral centre, each patient was evaluated in the outpatient clinic by a urologist. The assessment included a detailed review of the patient's history of urological surgeries, current bladder management strategy (clean intermittent catheterization [CIC], Valsalva voiding, urinary diversion or suprapubic catheter) and continence status.

A physical examination was conducted in the lithotomy position, assessing for stress urinary incontinence using a cough stress test, as well as for pelvic organ or rectal prolapse in female patients through a speculum examination of the vagina while asking the patient to strain and cough. The severity of cystocele, uterine prolapse, enterocele and rectocele was graded using the Baden‐Walker classification system.^9^

A urodynamic study was performed in all patients—except those with an ileal conduit—during their initial visit to the French referral centre and was repeated in some patients over time at the discretion of the urologist. The following urodynamic parameters—defined in accordance with ICS guidelines—were collected: cystometric capacity (CC, mL); volume at first sensation of bladder filling (mL); volume at first uninhibited detrusor contraction, if present (mL); bladder compliance, calculated manually from the traces (mL/cmH₂O); and maximum flow rate (Qmax, mL/s).

Renal function was assessed in all patients via a 24‐hour creatinine clearance test completed prior to their initial visit. As part of the institutional protocol, a CT urogram was performed in every patient at the time of their initial visit to establish a baseline morphological assessment of both the upper and lower urinary tract. Additionally, a cystoscopy was conducted in all patients—except those with an ileal conduit—during the initial evaluation, to screen for bladder stones and bladder cancer.

Urological follow‐up consisted of repeat visits every 1 to 2 years. At each follow‐up, the full clinical assessment was repeated, including evaluation of renal function and a urinary tract ultrasound. Urodynamic studies and cystoscopies were repeated selectively, at the discretion of the urologist.

Surgical failure was defined as either symptomatic recurrence of POP or anatomical recurrence (Baden–Walker grade ≥2) observed during follow‐up examination.

### 2.5 Outcomes of interest

The primary endpoint was the existence of a pelvic organ prolapse grade 2 or higher according to the Baden–Walker classification during the initial physical examination.

The secondary outcomes of interest were I) the need for surgery for symptomatic pelvic organ prolapse, II) recurrences of POP after surgery, III) the use of vaginal pessaries and IV) the symptoms associated with POP.

### 2.6 Statistical analysis

Means and standard deviations were reported for continuous variables, medians and ranges for categorical variables and proportions for nominal variables. Comparisons between groups were performed using the χ^2^ test or Fisher's exact test for discrete variables and the Mann–Whitney test for continuous variables as appropriate. Univariate and multivariate logistic regression analyses were performed to assess the risk factors of POP. Statistical analyses were performed using JMP pro v.18.0.1 software (SAS Institute Inc., Cary, NC, USA). All tests were two‐sided with *p* < 0.05 as a threshold to define statistical significance.

## 3 RESULTS

### 3.1 Patients' characteristics and POP prevalence

After exclusion of 41 patients under 18 years old or with other congenital abnormalities, 284 patients were included. The prevalence of POP was 14.8% (42 patients) and increased to 18.6% when grade 1 POP was taken into account (53 patients). The patients' characteristics are presented in Table 1. The median age was 33 years, and the vast majority of patients were nulliparous (75.8%). The type of spinal dysraphism was equally distributed, and the majority of patients were self‐catheterizing (57.9%). The prevalence of constipation was high (84.2%) The patients in the POP group were significantly older (median age: 44 vs. 31 years; p < 0.0001) with a higher proportion of patients with at least one childbirth (58.5% vs. 18.3%; p < 0.0001) although the proportion of nulliparous patients in the POP group remained surprisingly high (41.5%). There were also more patients with a history of cystectomy and ileal conduit in the POP group (11.9% vs. 3.8%; p = 0.04).

**TABLE 1:** Patients' characteristics.

	Whole cohort (N = 284)	POP (N = 42)	No POP (N = 242)	p‐value
Median age (IQR, years)	33 (25–45)	44 (35–45)	31 (23.8–42)	<0.0001
Mean BMI (kg/m^2^)	26.8 (+/−7.37)	26.8 (+/−7.7)	26.9 (+/−5.23)	0.50
Type of spinal dysraphism				
Open	120 (49.6%)	23 (60.5%)	97 (47.5%)	0.14
Closed	122 (50.4%)	15 (39.5%)	107 (52.5%)	
Level of neurological impairment				
Thoracic	6 (4.2%)	1 (3.5%)	5 (4.3%)	
Lumbar	113 (77.9%)	22 (75.9%)	91 (78.4%)	0.983
Sacral	26 (17.9%)	6 (20.7%)	20 (17.2%)	
Mobility status				
Ambulatory	162 (74%)	28 (75.7%)	134 (73.6%)	0.80
Weelchair user	57 (26%)	9 (24.3%)	28 (75.7%)	
Bladder management				
Spontaneous voiding	78 (33.5%)	12 (29.3%)	66 (34.4%)	0.81
CIC	135 (57.9%)	25 (61%)	110 (57.3%)	
Permanent drainage	20 (8.6%)	4 (9.8%)	16 (8.3%)	
Antimuscarinic intake	22 (21.6%)	6 (33.3%)	16 (19.0%)	0.18
Parity status				
Nulliparous	213 (75.8%)	17 (41.5%)	196 (81.7%)	<0.0001
At least one childbirth	68 (24.2%)	24 (58.5%)	44 (18.3%)	
History of cystectomy+ileal conduit	14 (5.1%)	5 (11.9%)	9 (3.8%)	0.04
Mean maximum cystometric capacity (ml)	433.8 (+/− 143.4)	429.2 (+/− 120.7)	434.9 (+/− 148.6)	0.82
Mean maximum urethral closure pressure (cmH_2_O)	66.6 (+/− 38.7)	45.9 (+/− 26.5)	77.2 (+/−39.8)	<0.0001
Mean Maximum detrusor pressure (cmH2O)	36.2 (+/− 55.9)	27.9 (+/−22.4)	37.2 (+/− 58.6)	0.76
Constipation	194 (84.2%)	35 (92.1%)	157 (82.6%)	0.11

* POP: pelvic organ prolapse grade 2 or higher according to Baden‐Walker classification.

* CIC: Clean intermittent catheterization.

### 3.2 Pelvic organ prolapse characteristics

The details of POP are summarized in Table 2. The predominant component was apical in 27 patients (64.3%), anterior in 10 patients (23.8%) and posterior in 5 (11.9%). There were two rectal prolapses (4.8%) and three grade 4 uterine prolapses (7.1%). All other POP were grade 2 or 3. Twenty‐one patients had a cystocele grade 3 or higher (7.4% of the whole cohort), 27 patients had an apical prolapse grade 2 or higher (9.5% of the whole cohort) and 10 had a rectocele/enterocele grade 2 or higher (3.5% of the whole cohort). The majority of patients had no symptoms deemed to be related to their POP (52.4%). Only five patients (11.9%) have reported the use of a pessary.

**TABLE 2:** Pelvic organ prolapse characteristics.

	N = 42
**Predominant component**	
Anterior	10 (23.8%)
Apical	27 (64.3%)
Posterior	5 (11.9%)
**Rectal prolapse**	2 (4.8%)
**Cystocele**	
Grade 0	29 (44.6%)
Grade 1	15 (23.1%)
Grade 2	14 (21.5%)
Grade 3	7 (10.8%)
**Apical prolapse**	
Grade 0	26 (40.6%)
Grade 1	11 (17.2%)
Grade 2	17 (26.6%)
Grade 3	7 (10.9%)
Grade 4	3 (4.7%)
**Rectocele/enterocele**	
Grade 0	46 (70.8%)
Grade 1	9 (13.9%)
Grade 2	9 (13.9%)
Grade 3	1 (1.5%)
Grade 4	0 (0%)
**Symptoms deemed related to the POP**	20 (47.6%)

### 3.3 Risk factors for POP

The univariate and multivariate assessment of POP predictors is shown in Table 3. The only factors that were significantly associated with POP were age (OR = 1.07; p < 0.0001), the parity status (OR = 6.29; p < 0.0001) and maximum urethral closure pressure (OR = 0.96; p < 0.0001). In multivariate analysis, adjusting for the type of spinal dysraphism, age and constipation, only the parity status (OR = 5.33; p = 0.005) and maximum urethral closure pressure (OR = 0.97; p = 0.02) remained significantly associated with the risk of POP.

**TABLE 3:** Predictive factors of pelvic organ prolapse in univariate and multivariate analysis.

Variables	POP							
	Univariate analysis				Multivariate analysis			
	Odds‐ratio	Confidence interval 95%		p‐value	Odds ratio	95% confidence interval		p‐value
		Lower	Upper			Lower	Upper	
**Type of spinal dysraphism**								
Open	1 [Ref]			‐	1 [Ref]			‐
Closed	0.59	0.29	1.19	0.14	0.31	0.08	1.03	0.06
**Mobility status**								
Ambulatory	1 [Ref]			‐	‐	‐	‐	‐
Wheelchair‐bound	1.11	0.51	2.65	0.79				
**Bladder emptying**								
Spontaneous voiding	1 [Ref]				‐	‐	‐	‐
Intermittent catheterization	1.25	0.60	2.73	0.56				
Permanent drainage	1.38	0.35	4.57	0.63				
**Maximum urethral closure pressure**	0.96	0.95	0.98	<0.0001	0.97	0.95	0.98	0.02
**Anticholinergic intake**	2.12	0.66	6.40	0.20	‐	‐	‐	**‐**
**Age**	1.07	1.04	1.09	<0.0001	1.04	0.99	1.10	0.07
**Constipation**	2.45	0.81	10.68	0.12	1.05	0.20	6.78	0.95
**Parity**								
Nulliparous	1 [Ref]			‐	‐	‐	‐	0.005
At least one childbirth	6.29	3.14	12.88	<0.0001	5.33	1.65	19.34	
**Body Mass Index**	0.98	0.94	1.05	0.98	‐	‐	‐	‐

### 3.4 Outcomes of POP surgical management

Eleven patients underwent surgical repair of their POP: five underwent sacrocolpopexy, three rectopexy and three native tissue transvaginal repair. Postoperative recurrence occurred in the majority of cases (n = 6, 54.5%), with half of these patients requiring a second or third surgery due to symptomatic recurrences occurring between 2 and 10 years after the initial procedure.

## 4 DISCUSSION

Women with spina bifida carry many possible risk factors for POP including a high prevalence of weak pelvic floor and Valsalva manoeuvres to empty their bladder or bowel. However, they also tend to have less childbirth than the general population, which may limit their risk of developing POP.^10^ In this study, we identified a significant prevalence of pelvic organ prolapse (POP) in young women with spina bifida, with apical prolapse as the predominant component. Most patients were nulliparous, findings that are consistent with those reported in previous studies.^11^ The parity status, along with maximum urethral closure pressure, appeared as the two risk factors associated with the occurrence of POP.

Our study design lacks a control group of women from the general population to confirm the higher prevalence of POP in women with spina bifida. To answer this question, a nationwide database would be required to compare the prevalence between patients with and without spina bifida. However, such study designs often come at the expense of fine clinical granularity and would not allow for an in‐depth assessment of potential risk factors and management outcomes. Comparing different series is always subject to numerous important drawbacks and could not be regarded as robust evidence. Although the prevalence of POP highly varied among existing cohorts,^12^ it is interesting to note that in the literature, the prevalence of POP ranges from 2.9% to 6.2% in women in their fourth decades.^13^, ^14^ Another strong argument to suggest the higher risk of POP in women with spina bifida is that 41.5% of POP occurred in nulliparous patients in our study while POP in nulliparous patients is a very uncommon occurrence in the non‐neurogenic population.^15^, ^16^

An interesting finding of the present study was the significant association between pelvic organ prolapse (POP) and low maximum urethral closure pressure (MUCP). Several hypotheses may explain this association. Pelvic floor fatigue and weakness are known contributors to both incontinence and POP^17^; however, limited data exist regarding the relationship between low MUCP or abnormal electromyography (EMG) and pelvic floor dysfunction in neurogenic patients, particularly those with spina bifida.^18^ We believe that low MUCP likely reflects a flaccid perineum or weak pelvic floor, as MUCP has been shown to correlate with pelvic floor muscle fatigability and weakness. Although POP may affect MUCP, it typically increases urethral pressure due to induced obstruction.^19^ This confounding factor was minimized in our study by reducing all prolapse prior to urodynamic testing. Finally, EMG of the striated perineal muscles during cystometry could support our hypothesis, as perineal fatigue has been associated with EMG changes^20^; however, this data was not available in our cohort.

The other risk factor, the parity status, was much more common. Unfortunately, the relatively limited sample size and inherent lack of statistical power prevented assessment of the impact of the type of delivery (vaginal vs caesarean section) on the risk of POP in the spina bifida population. One may still be tempted to favour C‐section in these patients to minimize the risk of POP.^21^ However, while the data on this topic in the general population remain controversial, no such data exist in spina bifida patients. Moreover, they often have undergone previous lower urinary tract surgical reconstruction (artificial urinary sphincter, augmentation cystoplasty, …) with an established increase in the risk of damaging these previous reconstructions in case of C‐section.^22^ Hence, the only action that could be drawn from the present analysis is to warn spina bifida patients of their increased risk of POP with childbirth and the need for careful per‐partum and post‐partum follow‐up.

Although the data available were limited, we observed a very high risk of recurrence after POP repair in our cohort. This finding would deserve further confirmation. However, one can hypothesize that the common occurrence of Valsalva voiding and weakness of the pelvic floor would determine this increased risk of recurrence. While further studies to assess the best possible management of POP in spina bifida patients are needed, clinicians should warn the patients of the high risk of recurrence after surgical repair and should address the reversible risk factors preoperatively by improving, for instance, bladder and bowel management to avoid Valsalva postoperatively.

Cystectomy and ileal conduit are well‐established risk factors of POP in women. In the present study, there were significantly more patients with a history of ileal conduit in the POP group. However, we could not assess this possible association in multivariate analysis due to a lack of statistical power (only five events of POP and a history of ileal conduit).

The present study has several limitations that should be acknowledged. Our definition of POP could be called into question as we decided to focus on prolapse grade +2 or higher.

Although the clinical relevance of prolapse stage 2 can be called into question in the general non‐neurogenic population, we believe that this threshold can be considered in spina bifida patients considering all the pelvic disorders that are going on in this population, which could be worsened or influenced by a stage 2 prolapse.

The Baden‐Walker classification is not without limitations. Indeed, it has been described in the literature as being subjective and lacking precision, with relatively poor interobserver and intraobserver reliability. The POP‐Q classification provides more robust and objective data and would have been better. However, during the study period (2007–2024), the Baden‐Walker classification was routinely used at our institution for all evaluations of pelvic organ support. The POP‐Q classification has been more recently adopted at our institution. In order to maintain consistency across the entire cohort and to ensure comparability within our dataset, we therefore retained the Baden‐Walker system in this analysis.

Imaging such as voiding cystourethrography, or MR defecography, could provide complementary and more objective assessments of POP. Unfortunately, they were not systematically performed for all patients in our cohort, as our institutional protocol relied primarily on clinical examination for initial POP assessment.

Moreover, the diagnosis was based only on a single physical examination, and one knows that the clinical evaluation of POP is subject to a variety depending on many factors. (morning vs afternoon, amount of physical exercise prior to the exam) Unfortunately, as our institution is a national referral centre, many patients undergo a comprehensive evaluation and are then followed at their respective university hospitals based on their main clinical issues, which explains why repeated examinations were not always available. Another significant limitation was the lack of adjustment for several possible risk factors of POP, such as Valsalva voiding or menopausal status, which could not be captured in this retrospective analysis of our database.^23^ As aforementioned, the relatively limited sample of patients with POP and inherent lack of statistical power prevented further investigation into several possible risk factors, such as a history of cystectomy + ileal conduit or the type of delivery (vaginal vs caesarean section), on the risk of POP in the spina bifida population.

## 5 CONCLUSION

In the present study, we found a high prevalence of POP grade 2 or higher in young adult women with spina bifida, with a high proportion of nulliparous women in those with POP. The parity status and a low maximum urethral closure pressure could be associated with an increased risk of POP in this population. The outcomes of surgical POP repair were poor with a high risk of recurrence. Our findings warrant careful information for spina bifida women on their increased risk of POP and routine screening of POP in these patients, as it could significantly impact their urinary, sexual and bowel symptoms as well as their management. Further studies are needed to determine the best preventive strategies and the best management of POP in spina bifida women.

## AUTHOR CONTRIBUTIONS

**Alexandre Dubois:** Conceptualization; data curation; writing—original draft; writing—review and editing. **Briac Malandain:** Data curation; writing—review and editing. **Juliette Hascoet:** Data curation; writing—review and editing. **Camille Haudebert:** Data curation; validation. **Charlène Brochard:** Data curation; validation. **Claire Richard:** Data curation; validation. **Caroline Voiry:** Data curation; validation. **Krystel Nyangoh Timoh:** Data curation; validation. **Andrea Manunta:** Data curation; validation. **Emmanuelle Samson:** Data curation; validation. **Benoit Peyronnet:** Conceptualization; data curation; investigation; resources; supervision; validation; visualization; writing—original draft; writing—review and editing.

## CONFLICT OF INTEREST STATEMENT

Benoît PEYRONNET is consultant for Boston Scientific, Intuitive, Medtronic, Pierre Fabre, Ibsa, Coloplast, Schwa Medico, Hollister, Ipsen, AbbVie.

Other authors have nothing to disclose.

## Data Availability

The data that support the findings of this study are available on request from the corresponding author. The data are not publicly available due to privacy or ethical restrictions.
